# Reliable mortality statistics for Turkey: Are we there yet?

**DOI:** 10.1186/s12889-015-1904-1

**Published:** 2015-06-10

**Authors:** Raziye Özdemir, Chalapati Rao, Zeliha Öcek, Gönül Dinç Horasan

**Affiliations:** Department of Occupational Health and Safety, Karabuk University Health School, Karabuk, Turkey; Research School of Population Health, Australian National University, Canberra, Australia; Department of Public Health, Ege University Medicine Faculty, Izmir, Turkey; Department of Biostatistics and Medical Informatics, Celal Bayar University Medicine Faculty, Manisa, Turkey

**Keywords:** Turkish Statistical Institute, Death Reporting System, Vital Registration, Mortality statistics, Izmir, Turkey

## Abstract

**Background:**

The Turkish government has implemented several reforms to improve the Turkish Statistical Institute Death Reporting System (TURKSTAT-DRS) since 2009. However, there has been no assessment to evaluate the impact of these reforms on causes of death statistics. This study attempted to analyse the impact of these reforms on the TURKSTAT-DRS for Turkey, and in the case of Izmir, one of the most developed provinces in Turkey.

**Methods:**

The evaluation framework comprised three main components each with specific criteria. Firstly, data from TURKSTAT for Turkey and Izmir for the periods 2001–2008 and 2009–2013 were assessed in terms of the following dimensions that represent quality of mortality statistics (a. completeness of death registration, b. trends in proportions of deaths with ill-defined causes). Secondly, the quality of information recorded on individual death certificates from Izmir in 2010 was analysed for a. missing information, b. timeliness of death notifications and c. characteristics of deaths with ill-defined causes. Finally, TURKSTAT data were analysed to estimate life tables and summary mortality indicators for Turkey and Izmir, as well as the leading causes-of-death in Turkey in 2013.

**Results:**

Registration of adult deaths in Izmir as well as at the national level for Turkey has considerably improved since the introduction of reforms in 2009, along with marked decline in the proportions of deaths assigned ill-defined causes. Death certificates from Izmir indicated significant gaps in recorded information for demographic as well as epidemiological variables, particularly for infant deaths, and in the detailed recording of causes of death. Life expectancy at birth estimated from local data is 3–4 years higher than similar estimates for Turkey from international studies, and this requires further investigation and confirmation.

**Conclusion:**

The TURKSTAT-DRS is now an improved source of mortality and cause of death statistics for Turkey. The reliability and validity of TURKSTAT data needs to be established through a detailed research program to evaluate completeness of death registration and validity of registered causes of death. Similar evaluation and data analysis of mortality indicators is required at regular intervals at national and sub-national level, to increase confidence in their utility as primary data for epidemiology and health policy.

**Electronic supplementary material:**

The online version of this article (doi:10.1186/s12889-015-1904-1) contains supplementary material, which is available to authorized users.

## Background

Death statistics from vital registration (VR) are valuable data sources for monitoring the health of populations and for setting priorities [1, 2]. There is a critical need for reliable VR data on mortality in the majority of developing countries [3–5]. This includes Turkey which was ranked among the countries with VR data of limited use in an international assessment conducted in 2007 [5]. Previous analyses identified significant problems with mortality data in Turkey, including incomplete registration, missing variables, and errors in reporting or classification of causes-of-death [5–9], Hence, the utility of available Turkish mortality statistics for public health purposes remains questionable [5].

In the absence of high quality national empirical mortality data, mortality estimates for Turkey were developed for the year 2000 as part of the Turkish National Burden of Disease and Cost Effectiveness Project (NBD-CEP) in 2004. These estimates were derived using demographic and epidemiological models, which relied on various adjustments to biases in empirical local data on deaths by age, sex and cause, obtained from vital registration in urban areas in Turkey in 2000 [10]. There were two key adjustments. Firstly, a correction factor of 20 % as estimated from indirect demographic techniques was applied to adjust incomplete death registration in urban areas. Secondly, a generic algorithm was applied to reallocate of over 40 % of deaths that had been assigned to ill-defined causes in VR data to specific cause of death. These generic reallocation algorithms followed standard design as developed in the Global Burden of Disease (GBD) 2000 study [11].

For rural areas, comprising 35 % of Turkish populations at that time, estimates were separately derived using a combination of model life-tables and cause of death models, based on the estimated urban mortality patterns as described above. Although these final Turkish mortality estimates were based on the best available information at that time, the underlying rationale and methods indicate that these estimates are only weakly anchored in local data.

Little attention had been paid to improve the death registration in Turkey in the period immediately after the NBD-CEP, i.e. from 2004 to 2009. It was only in 2009 that government initiatives were conducted to improve the Turkish Statistical Institute Death Reporting System (TURKSTAT-DRS), described below. This article presents findings from an evaluation of mortality statistics from TURKSTAT-DRS for Turkey as well as Izmir province during the period 2000–2013, to understand overall changes in the quality of mortality data as a result of these reforms. We also present an analysis of information from all individual death certificates from Izmir province in 2010, in order to understand the quality of documentation practices in regard to death certification. Given that Izmir is among the better developed provinces in Turkey [12], we believe that data from Izmir is likely to represent the higher end of the range of mortality data quality across Turkey. Findings from this evaluation will guide interventions to further strengthen the availability and quality of Turkish mortality statistics.

### Death registration in Turkey

**Fig. 1:**
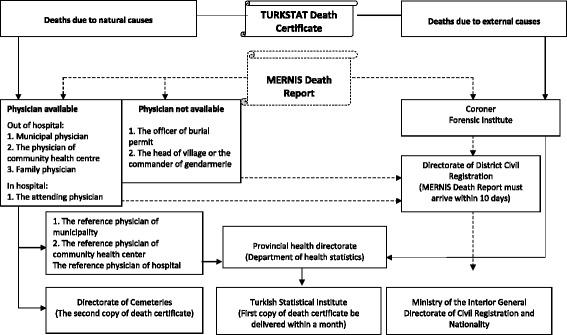
Generic reporting documentation completed for death registration and statistical compilation in Turkey

A brief description of the design of the death registration systems and mechanisms for compilation of vital statistics in Turkey, along with some details on the reforms instituted in 2009 is useful to place this research into context. There are two main organizations responsible for administration of death records in Turkey [Fig. 1). The first organization is the Civil Registration System under Ministry of Internal Affairs which is only responsible for the official process of death registration. Under the Civil Registration System, death events for the whole country are recorded through the Central Population Administrative System (MERNIS) Reporting Form. Data from the MERNIS forms are only meant for administrative purposes, and include a single line for recording the causes of death. In rural areas without health practitioners, the MERNIS form is the only reporting practice implemented, and deaths are registered by village headmen, with lay reported cause of death. All unnatural deaths in Turkey are reported to the coroner. Data from all MERNIS forms are compiled into national data by the Ministry of Internal Affairs.

The second organization responsible for compiling and disseminating death statistics is the TURKSTAT-DRS. In practice, the TURKSTAT-DRS only covers urban areas, and implements a death certificate independent of the MERNIS report. When a death occurs in a health facility, the attending physician completes the TURKSTAT Death Certificate, which also includes a provision for reporting causes of death. For deaths in urban areas that occur at home, the death certificate is completed by municipality or primary health care physicians. The certificate is completed with three copies, the first copy submitted to the provincial public health directorates, the second copy given to a relative of the deceased as an authorization for burial, and the third copy retained at the issuing institution. At the Izmir provincial health directorate, an electronic data base compiles all variables from each received death certificate. Subsequently, all hard copies of received death certificates are eventually submitted to TURKSTAT.

It has been widely perceived that prior to the reforms, both the MERNIS and TURKSTAT-DRS did not function efficiently and records from both systems remained insufficient for statistical purposes [13–16]. The reforms implemented throughout Turkey in 2009 have brought changes from four aspects. Firstly, instructions have been issued to improve coordination between different agencies responsible for vital registration at local level, to ensure completeness of records. Secondly, there have been initiatives to implement routine procedures to reconcile death records collected by the MERNIS and TURKSTAT-DRS [17]. This is very important to enable compilation of comprehensive national mortality data. Hence, since 2009, the TURKSTAT database includes all reported deaths by merging the data from MERNIS reports as well as from death certificates submitted by the Provincial Health Directorates to TURKSTAT. Thirdly, the previous version of the TURKSTAT Death Certificate till 2008 (see Additional file 1) contained limited variables and allowed the recording of only a single cause of death, but has now been adapted to international standards for recording multiple causes of death [18]. The current revision in place since 2009 in includes two parts (see Additional file 2); Part-I is for reporting a chain of events leading directly to death, and Part-II is for reporting all other significant diseases, conditions, or injuries that contributed to death. The underlying cause of death must appear on the lowest completed line of Part-I in accordance with the general principle of the international rules [18]. In addition, the current version also added variables to record in detail the place of death, manner of death (natural causes/injury/suicide/homicide), specific information pertaining to maternal and perinatal mortality, among others. All these changes have resulted in a well-designed and comprehensive death certificate. Finally, causes of death are now classified and coded according to The Tenth Revision of the International Statistical Classification of Diseases and Related Health Problems (ICD-10), instead of the Eight-Revision of the ICD used till 2008.

Currently, the TURKSTAT website makes publically available the following data each year [17, 19]:

- Annual number of deaths by sex and five-year age groups for each district and province
- Monthly and annual numbers of infant deaths by district and province
- Deaths by single age for Turkey
- Deaths by age group and marital status for Turkey
- Annual numbers of deaths by sex and cause for Turkey according to a selected list of 89 causes
- Annual numbers of deaths for Turkey by sex and broad age groups (0–14, 15-24…,85+) according to seven ICD chapter groups (Circulatory system; Neoplasms; Respiratory System; Endocrine, nutritional and metabolic diseases; Injuries; Nervous System, and All other causes)
- Annual numbers of deaths for each province according to above mentioned seven broad cause groups

More detailed data compilations are only available at the provincial level, including information on detailed causes of death, based on compilation of death certificates submitted to TURKSTAT by provincial health directorates.

A summary of the chronological development of death registration practices of TURKSTAT-DRS along with various reforms at different time points is presented in Additional file 3.

It is important to undertake a comprehensive evaluation after the introduction of these reforms, in order to assess their impact on the quality of mortality statistics, and identify areas which need continued or additional attention. In view of the availability of detailed mortality data for Turkey and Izmir from the TURKSTAT database for the period 2000–2013, we conducted a comparative analysis of completeness of death registration and trends in proportions of deaths assigned ill-defined causes before and after the reforms. Also, we obtained access to detailed individual death certificate data from Provincial Health Directorate of Izmir for 2010, and present the findings on the quality of data from death certificates in 2010 as a baseline for future assessments.

## Methods

### Study setting

This study was conducted at the national level for Turkey with a population of 74 million in 2010, and at the sub national level in Izmir (western Turkey) which is one of the most socioeconomically developed provinces in the country. Izmir has a population of about four million, 5.4 % of Turkey population, in 2010 [20].

### Data sources

Several data sources were used for the various analyses presented in this paper. For the assessment of completeness of death registration using indirect demographic techniques, the TURKSTAT database on deaths in Turkey and in Izmir by age, sex and five-year age groups for each year from 2001 to 2013 was accessed. Population denominators for this analysis were taken from 2000 general census, and from the TURKSTAT Address Based Population Registration System (ABPRS) for 2008, 2009, and 2013 [20]. Finally, for analyzing the quality of individual variables recorded on the death certificates, we accessed the electronic database of all certificates collected at the Izmir Provincial Health Directorate (n = 18933). An official permission was received to use the data from Izmir Provincial Health Directorate (No. 990/18755). Also the data not openly available from TURKSTAT website was provided by individual application to TURKSTAT.

## Analytical framework and methods

### Assessment of quality of mortality statistics for Turkey and Izmir province, 2001–2013

The quality of mortality statistics was assessed across two broad dimensions; one related to the generalizability of the data, in terms of completeness of death registration; and the second an assessment of the validity of reported causes of death, in terms of the proportion of deaths assigned ill-defined causes [4, 21].

#### Completeness of death registration

A comparative analysis of completeness of death registration between periods 2001–2008 & 2009–2013 was conducted for Turkey as well as Izmir, to assess the impact of TURKSTAT reforms. Completeness was defined as the estimated proportion of adults (≥5 years of age) deaths occurred in Izmir being reported to TURKSTAT. This proportion was determined using the combination of the Generalized Growth Balance (GGB)-the Synthetic Extinct Generations (SEG) methods, which reduces the potential for bias [22, 23]. This combined method produces an estimate of completeness across each of the reference periods mentioned above.

The method was implemented in two stages. In the first stage, the relative completeness of the 2000 population census counts, as well as the population data for 2008, 2009, and 2013 from ABPRS for Izmir and Turkey were assessed by comparing the age distributions observed in them using the GGB method. Appropriate corrections to population counts were made following this assessment, as described in Table 1. In the second stage, the completeness of adult death registration was calculated according to the SEG method by using standardized population counts.

**Table 1:** Summary of estimated population and death registration completeness (%) for Turkey and Izmir, during the periods 2001–2008 and 2009–2013

Study population	Population		Death registration	
	2001-2008	2009-2013	2001-2008	2009-2013
Turkey				
Male	93.5	99.5	57.7	99.2
Female	95.3	99.6	61.1	97.0
Izmir				
Male	95.4	99.7	80.0	103.9
Female	94.2	97.8	72.4	102.0

#### Proportions of ill-defined causes of death

As mentioned earlier, the format of the new TURKSTAT Death Certificate is aligned with international norms for reporting causes of death [18]. Underlying causes for all deaths are coded according to ICD-10. For data evaluation according to this criterion, the proportion of deaths assigned to the ICD codes for symptoms, signs, and ill-defined conditions (R00-R99); and cardiovascular disease categories lacking diagnostic meaning (ICD-10 codes I46, I47.2, I49.0, I50, I51.4, I51.5, I51.6, I51.9, I70.9) These categories were applied to assess the overall proportions of ill-defined deaths in TURKSTAT data for Izmir & Turkey, for the annual time series from 2000–2013 to assess the impact of TURKSTAT reforms on data quality in this dimension.

#### General characteristics of registration data from death certificates from Izmir, 2010

The quality of general characteristics of registration data from death certificates compiled by the Izmir Provincial Health Directorate for 2010 was assessed across three dimensions, as a baseline for future assessments of the quality of death certification. Firstly, **missing information** on key variables of death certificates was assessed, particularly in regard to demographic characteristics of the deceased, as well as for variables pertaining to infant mortality and deaths from injuries. The quality of recorded causes of death was assessed in terms of the proportions of deaths with no cause, or ordinal values of numbers of recorded causes. Secondly, the **timeliness of notification** was assessed in terms of proportions of deaths registered within or beyond 30 days of the occurrence. The deaths in each of the two categories of timeliness were analyzed according to characteristics such as residential area, place of death, and reporting institution, among others. Finally, the **characteristics of deaths with ill**-**defined causes** at initial death certification was assessed particularly in terms of gender, age, manner of death, and reporting institution, among others.

### Analysis of mortality indicators

#### Life tables and summary mortality indicators

Life tables for reported and adjusted deaths were constructed by sex for 2013 using conventional Abridged Life Table Method [24]. Adjusted death numbers for life tables were obtained through standardization of reported death numbers according to estimates of completeness by sex from GGB-SEG methods for the periods 2000–2008 & 2009–2013 for Izmir & Turkey. Standard definitions were applied to compute summary indicators such as risks of under-five mortality and adult mortality (between ages 15 and 60 years) as recommended by the World Health Organization (WHO) [24]. These finding were compared with similar estimates derived for Turkey by the WHO for 2012 [25], and the GBD Study for 2013 [26].

#### *Analysis of leading* cause of death for Turkey, 2013

As mentioned earlier, data from TURKSTAT are available in different ICD code aggregations, at national and district level. We present the findings on the twenty specific leading causes of death by sex for Turkey in 2013, as derived from the available selected list of causes of death.

## Results

### Assessment of quality of mortality statistics

#### Comparative analysis of completeness of death registration

As can be noted from Table 1, the SEG-GGB analysis involved minor adjustments to population counts in both periods, and relatively substantial corrections to deaths at the national level as well as for Izmir, during the period 2001–2008. The findings for the period 2001–2008 suggest that there was little change in death registration completeness when compared with previous such assessments conducted on data of about a decade ago, for the Turkish NBD-CEP [6]. However, the analysis for the period 2009–2013 indicates that the completeness of adult death registration has considerably improved in both study populations, possibly due to introduction of collaboration between the MERNIS and TURKSTAT data compilations since 2009. Detailed analysis indicates that there is very little variation in completeness across age groups. However, known limitations of indirect methods in assessing completeness [22] necessitate that these estimates of completeness should be interpreted with caution, and these analysis should be repeated at regular intervals, as well as triangulated with other methodologies for completeness assessment such as ‘capture-recapture’ analysis.

#### Trend in proportions of deaths with ill-defined causes

The trend in the quality of recorded causes of death for the study period, as assessed by the proportions of deaths assigned ill-defined causes, mirrors the findings from the assessment of completeness of registration, in terms of a marked improvement since the introduction of the 2009 reforms. A key factor responsible for this development is a specific initiative launched by TURKSTAT to address this issue.^1^ As mentioned earlier, copies of all death certificates are routinely submitted by provincial health directorates to TURSKSTAT. Each year, TURKSTAT returns all death certificates that have been assigned causes coded to the ill-defined categories back to the provincial health directorates, for verification and or correction of the cause, by the respective hospital, municipality, or primary care certifying physicians who initially certified the death. While the exact process implemented for verification or correction of the cause is not clarified, the updated death certificates are directly returned to TURKSTAT, without any changes made to the records maintained at the province level. Overall, the final results published by TURKSTAT are indeed impressive in this dimension, but further research is required to understand the exact details of the verification procedures, and with more accurate quantification of the impact of various reforms. In particular, the evaluation of death certificates from Izmir, as described in Table 4 and accompanying text in the following section, provides additional context on this issue.

### Assessment of quality of general variables

#### Missing information

Most demographic variables were adequately entered on death certificates, except in the case of educational status and profession, for which information was missing on 44 % of certificates. This could limit any analyses of mortality according to these social determinants. Also, in almost all cases, the variable to identify deaths from injuries (Yes or No) was left blank. While the quality of information on variables pertaining to deaths such as the place of death is complete, this was not so in the case of infant deaths. In about 83 % of infant deaths the actual time of birth was not recorded, and in over a quarter of such events the birth weight and duration of gestation were missing. The absence of these data limits secondary analysis to understand the determinants of infant mortality. In regard to the recording of causes of death, approximately 32 % of certified deaths were attributed to a single cause, 41 % to a sequence of two causes, and only a quarter of all deaths had a sequence of three or more causes. Also, the section of contributing cause of death was blank for nearly all certificates [Table 2]. It should be borne in mind that these findings were based on analysis at initial death certification. The actual status in this dimension, after the completion of verification and correction protocols for deaths with ill-defined and non-specific cardiovascular codes, is not known. However, these limitations at the stage of initial stage of certification are considerable, and should be assessed through a repeat analysis in the near future, to identify any improvements in this dimension.

**Table 2:** The proportion of missing information in death certificates from Izmir, 2010

Variables	Frequency of missing information	
Sociodemographic characteristics of decedent (n = 18933)	n	%
The level of education	8325	44.0
Profession	8251	43.6
The place of birth	3229	17.1
Sex	31	0.2
Residential area	42	0.2
The date of birth	27	0.1
The date of death	0	0.0
Variables associated with death (n = 18933)		
Did the death occur as a result of injury?	18186	96.1
The time of death	1063	5.6
Was an autopsy performed?	602	3.2
Place of death	370	2.0
Manner of death	202	1.1
Variables associated with stillbirths and infant deaths (n = 343)		
The time of birth	285	83.1
The sequence of birth	142	41.4
Birth weight	93	27.1
The age of the mother	97	28.3
Gestation in weeks	102	29.7
The cause of death (n = 18933)		
Causative chain		
a line	433	2.3
b line	6441	34.0
c line	14190	74.9
d line	17849	94.3
The contributory cause of death		
a line	17960	94.9
b line	18701	98.8

#### The timeliness of death registration

Only one-thirds of deaths were registered at Provincial Health Directorate within 30 days after the death event, and this proportion applies to deaths registered by hospitals as well as deaths initially reported to the community health centres. This suggests that there is need for improved collaboration between the health system and TURKSTAT for more timely data transfer [Table 3]. Even in the case of deaths from external causes, more than 50 % are affected by delayed registration, indicating the need for improved collaboration between TURKSTAT and the coronial and police death recording systems. The delay in notification of deaths for which an autopsy was performed is plausible, in view of the time taken to confirm the cause of death. However, the all poor timeliness indicates limited enforcement of the death reporting legislation, and such limited enforcement may also be a factor that results in non-reporting of events and therefore incompleteness of death registration.

**Table 3:** The notification duration of deaths according to demographic and medical variables on death certificates from Izmir, 2010

Variables	Notification duration			
	≤30 days		>30 days	
	n	%	n	%
Sex				
Male	3753	35.7	6757	64.3
Female	2807	33.3	5612	66.7
Unknown	-	-	3	100.0
Residential area				
Metropolitan district	4778	35.3	8761	64.7
Countryside district	1767	33.0	3584	67.0
Unknown	15	35.7	28	64.3
The place of death				
Home	2337	29.6	5554	70.4
Hospital	3799	38.7	6026	61.3
Other^a^	312	36.8	535	63.2
Unknown	112	30.3	258	69.7
Manner of death				
Natural	6107	34.4	11643	65.6
Due to external causes	231	44.2	292	55.8
Other^b^	178	38.9	280	61.1
Unknown	44	21.8	158	78.2
Was an autopsy performed?				
Yes	381	42.6	514	57.4
No	5940	34.1	11494	65.9
Unknown	239	39.6	365	60.4
Reporting institute				
Institute of Forensic Medicine	382	43.2	502	56.8
Municipality	1547	29.7	3666	70.3
Hospital	3755	38.3	6041	61.7
Community health center	690	30.4	1578	69.6
Other^c^	183	24.0	579	76.0
Unknown	3	30.0	7	70.0
Total	6560	34.6	12373	65.4

^a^The death places of work place, ambulance, other vehicle and other in death certificates

^b^The manner of death of other, pending investigation and unknown in death certificate

^c^Private medical centers, private policlinics, health directorates of other provinces except Izmir and homes for the elderly

#### Characteristics of deaths assigned ill-defined causes

Table 4 displays the characteristics of death certificates in Izmir, in terms of their propensities to be certified with ill-defined causes. It should be noted that this table represent the initial certified diagnosis, as opposed to the final diagnoses that are determined after the verification/correction protocols described in the earlier section on trends of ill-defined causes. Presentation of these findings serves as a baseline assessment for future evaluation of the quality of death certification and diagnoses.

**Table 4:** Characteristics of death certificates from Izmir with defined and ill-defined underlying causes of death, 2010

	Underlying cause of death			
Variables	Defined		Ill-defined	
	n	%	n	%
Sex				
Male	7823	77.0	2336	23.0
Female	5924	71.0	2417	29.0
Age groups				
0-14	344	72.9	128	27.1
15-29	213	77.7	61	22.3
30-44	462	79.1	122	20.9
45-59	2078	80.6	500	19.4
60-74	4412	78.7	1197	21.3
75+	6224	69.4	2743	30.6
Unknown	14	87.5	2	12.5
Residential area				
Metropolitan district	9804	73.9	3455	26.1
Countryside district	3915	75.2	1289	24.8
Unknown	28	75.7	9	24.3
Manner of death				
Natural	13120	74.2	4571	25.8
Due to external causes	390	84.1	74	15.9
Other^b^	105	62.9	62	37.1
Unknown	132	74.2	46	25.8
Reporting institute				
Institute of Forensic Medicine	385	82.1	84	17.9
Municipality	3567	68.5	1637	31.5
Hospital	7423	75.8	2368	24.2
Community health center	1784	78.7	484	21.3
Other^c^	580	76.5	178	23.5
Unknown	8	80.0	2	20.0
Was an autopsy performed				
Yes	401	82.2	87	17.8
No	12931	84.2	4488	25.8
Unknown	415	70.0	178	30.0
Total	13747	74.3	4753	25.7

^a^The death places of work place, ambulance, other vehicle and other in death certificates

^b^The manner of death of other, pending investigation and unknown in death certificate

^c^Private medical centers, private policlinics, health directorate of other provinces except Izmir and homes for the elderly.

Based on these initial diagnoses, nearly one-thirds of cause of deaths (29.7 %) were not usable for public health purposes, and about 60 % of these 5621 deaths with ill-defined or unknown causes had been assigned non-specific cardiovascular causes (data not shown).

The proportion of ill-defined cause of death were observed higher for females, for deaths which occurred in home, for deaths due to external causes, and for deaths for which autopsy had been performed [Table 4]. Also, in regard to the age distribution, it is noted that about a third of all deaths in the 15–59 year age groups are coded to ill-defined conditions; which is a matter of concern since this is the age group where mortality is largely from preventable causes. While the introduction of the verification/correction protocols may have minimized this issue, there need to be further evaluation of this aspect of death certification, preferably through a comprehensive research study similar to those conducted in other settings [27, 28].

### Analysis of mortality indicators

#### Reported and adjusted life-tables and summary mortality indicators

The estimated summary mortality indicators for Izmir and Turkey from TURKSTAT data in Table 5 demonstrate considerably lower mortality patterns than the international estimates for Turkey, as derived by the World Health Organization and the GBD 2013 Study [25, 26]. These findings are largely driven by the lower estimates of risks of adult mortality, in both males and females, from the two international data sources. The differences in life expectancy at birth ranges from 3–5 years for males and females across the three sources of estimates for Turkey, which raises concerns about differences in estimation methodologies. Also, while the total number of deaths for females estimated in this study (172233) are higher than the estimates from the GBD 2013 study (147916), the life expectancy at birth for females is higher from this study, indicating there are also notable variations in age-specific mortality rates between the different sources. The national estimates of under-five mortality derived from these analyses closely resemble the estimate of 15 per 1000 live births derived from the Turkish Demographic and Health Survey of 2013 [29]. Overall, the findings in Table 5 indicate an increase of at least 5 years in the life expectancy at birth in both males and females, since the previous estimates for Turkey in 2000 (males - 67.7 years, females - 71.9 years) [10], reflecting considerable improvements in population health status over the past decade.

**Table 5:** Summary mortality indicators for Izmir and Turkey from TURKSTAT compared with estimates from WHO and GBD Study, 2013

	Adjusted estimates for Izmir, 2013		Adjusted estimates for Turkey, 2013		WHO estimates for Turkey, 2012^a^		GBD Study estimates for Turkey, 2013	
Indicator	Males	Females	Males	Females	Males	Females	Males	Females
Total deaths	12252	9872	206661	172233	237306	184415	214907	147916
Life expectancy at birth (in years)	77.7	84.5	76.4	82.4	72.0	78.0	72.9	79.6
Life expectancy at age 60 (in years)	21.8	27.0	21.1	25.6	18.0	23.2	19.2	23.4
Risk of under-five mortality (per 1000 live births)	9.2	8.4	14.5	13.4	15.3	13.1	17.4	17.4
Risk of adult mortality^b^ (per 1000 population)	99.4	44.6	105.3	51.4	150.0	75.0	150.0	60.0

^a^Source: World Health Statistics 2013 ^b^between ages 15 and 59 years

#### Analysis of leading cause of death

**Fig. 2:**
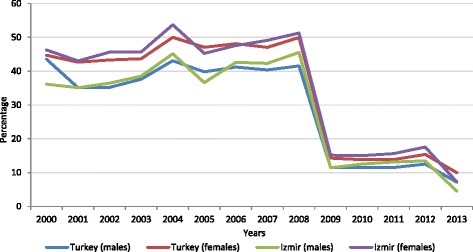
Trends in proportions of deaths assigned to ill-defined causes* in Izmir and Turkey, 2000–2013. *Codes for ill-defined causes listed in Methods section.

Table 6 presents the 20 leading causes of death by sex for Turkey, derived from the TURKSTAT selected list of causes of death available for 2013. The sharp decline in proportions of deaths assigned ill-defined causes since the introduction of TURKSTAT reforms in 2009 (Fig. 2) has resulted in a clear identification of the specific priority conditions responsible for premature mortality in Turkey. As can be observed from Table 6, the leading causes are dominated by non-communicable diseases, particularly cardiovascular conditions and cancers, with only pneumonia in both males and females, along with transport accidents in males and accidental falls in females as the exceptions. This represents considerable progression through the epidemiological transition since the previous estimates for the year 2000 [6] in which diarrhoeal disease and tuberculosis were also estimated among the leading causes of death. The burden from lung cancer and chronic obstructive pulmonary disease in males also reflects the impact of the smoking epidemic in Turkey.

**Table 6:** Leading causes of death by sex in Turkey in 2013

	Males			Females		
Rank	ICD-10 codes	Cause of death	Percentage	ICD-10 codes	Cause of death	Percentage
1	I20-I25	Ischemic heart diseases	16.4	I20-I25	Ischemic heart diseases	14.3
2	C32-C34	Lung cancer	10.2	I60-I69	Cerebrovascular diseases	12.3
3	I60-I69	Cerebrovascular diseases	8.2	I39-I52	Other heart diseases	8.6
4	J40-J47	Chronic lower respiratory diseases	7.6	I10-I15	Hypertensive diseases	7.0
5	I39-I52	Other heart diseases	5.7	E10-E14	Diabetes mellitus	5.9
6	I10-I15	Hypertensive diseases	3.6	J40-J47	Chronic lower respiratory diseases	5.4
7	E10-E14	Diabetes mellitus	3.5	G30	Alzheimer disease	3.3
8	V01-V99	Transport accidents	2.6	N17-N19	Renal failure	2.8
9	N17-N19	Renal failure	2.3	R00-R99	Ill-defined causes	2.3
10	C16	Stomach cancer	2.2	C50	Breast cancer	2.2
11	R00-R99	Ill-defined causes	2.1	C32-C34	Lung cancer	2.0
12	P00-P96	Perinatal conditions	2.0	J12-J18	Pneumonia	1.9
13	G30	Alzheimer disease	1.9	P00-P96	Perinatal conditions	1.8
14	C61	Prostate cancer	1.8	C81-C96	Blood cancers	1.6
15	C81-C96	Blood cancers	1.8	Q00-Q99	Congenital malformations	1.4
16	J12-J18	Pneumonia	1.8	C16	Stomach cancer	1.4
17	C18	Colon cancer	1.5	C18	Colon cancer	1.3
18	C25	Pancreatic cancer	1.3	C25	Pancreatic cancer	1.1
19	Q00-Q99	Congenital malformations	1.3	W00-W19	Accidental falls	1.1
20	W00-W19	Accidental falls	1.0	V01-V99	Transport accidents	1.0
		All other causes	21.2		All other causes	21.1
		Total	100.0		Total	100.0

Source: Turkish Statistical Institute causes of death statistics [16]

## Discussion

This assessment of mortality data in Turkey demonstrates considerable improvement over the past decade, in the two important dimensions of completeness of death registration as well as specification of causes of death. The reforms to the tools and procedures for death certification and notification as well as in compilation and processing of data, as introduced by TURKSTAT in 2009, have been instrumental in the accomplishment of these objectives. The high levels of data completeness as estimated by this study as well as by the GBD 2013 study create confidence in the use of TURKSTAT data to derive summary mortality measures as well as to assess the magnitude of leading causes of death [30]. Ideally, all-cause and cause-specific mortality rates for the population can be reasonably estimated at registration completeness levels of at least 90 % [31, 32], and these levels appear to have been achieved in Turkey, at least according to the analyses presented here, as well as the estimates of completeness for Turkey derived for the GBD 2013 study. Another important development has been the achievement of comparable levels of completeness for males and females, suggesting uniformity in the implementation of legal protocols and societal compliance with death registration.

The differences in estimates of summary mortality indicators across different sources could be due to several reasons. Some degree of variation can be expected from use of different sources of population data, as well as in time points for the deaths -the WHO estimates are based on the United Nations Population Division [33] analysis of TURKSTAT mortality data for 2009, while the GBD 2013 estimates use the TURSKTAT 2011 mortality data. The differences in population estimates could also be affected by unmeasured population migration. There is also variation in the completeness estimates and resultant correction factors applied -the GBD Study estimates completeness of TURKSTAT 2011 data using the SEG/GGB methods to be 91 % [26],^2^ while there is no mention of the estimated completeness/correction factors applied to the WHO estimates. The completeness of death registration in Izmir is estimated to be higher than the national level, as per expectation. While there has been consistent improvement in completeness of death registration in Turkey in recent times [34], the current levels need to be confirmed. In summary, the overall differences in life expectancies of 3–5 years between the different estimates warrant a thorough comparative analysis of the data sources and methods used in such demographic estimation exercises, in order to generate a consensus interpretation on population and mortality estimates for Turkey. Also, given the limitations of indirect demographic techniques [22], efforts should be made to conduct direct assessments using ‘capture-recapture’ methods, as an alternate means to assess completeness of death registration [35].

Inaccuracies and errors in the completion of death certificates can lead to biased estimation of several epidemiological parameters [30, 36, 37]. In this study, we noted that significant proportions of key variables important for secondary analyses of mortality data were missing on death certificates. This could be a result of either genuine lack of availability of information, or inadequate attention to the quality of information being recorded on them. This aspect should be emphasized in training programs for death certification. Further research is required to identify the specific reasons for such missing information, and design interventions to improve the quality of death certificates.

In regard to recorded causes of death, it was seen from the analysis of death certificates in Izmir that the majority of deaths were attributed to only one or two causes, and there was no importance given to causality chain, and the listing of contributory causes of death. Since most deaths in Izmir occur among adults, there is likely to be considerable co-morbidity from chronic disease. There is also potential for a chronological and pathophysiological sequence of clinical events prior to death, which should be documented accurately. These factors result in an expectation of higher proportions of deaths with multiple cause sequences in Part 1, as well as mentions of co-morbidities on the death certificates. Training programs on cause of death certification could emphasize the need for more detailed listing of causes of death.

Currently, TURKSTAT has started to compile data from death certificates in electronic formats since the beginning of 2013, and it can be anticipated that there will be a decline in proportions of incomplete and inaccurate notifications in the future.

The actual magnitude of mortality from various causes may be more clearly understood if there is greater accuracy in the specification of causes for deaths currently assigned codes from the ‘other heart diseases’, as well as from hypertensive diseases. The findings from the death certificates for Izmir identify that about 20 % of all deaths are assigned cardiac arrest, cardiorespiratory insufficiency and heart failure, among other such ill-defined cardiovascular causes, at initial certification. Previous studies in Turkey investigating this issue showed 65-90 % of individuals assigned such ill-defined cardiovascular conditions as the cause of death did not have any heart disease during their lifetime [38, 39].

This should be further investigated when reviewing the TURKSTAT protocols for verification of ill-defined causes, to identify the empirical basis for correction of the cause of death on individual death certificates, particularly for ill-defined cardiovascular conditions. In summary, these findings on specified causes of death in registration data need to be substantiated by detailed research studies to validate cause attribution, at least in a representative sample of deaths with defined as well as ill-defined causes. This is required for community deaths registered in municipalities as well as community health centres, that occur in the absence of medical attention [27]. A detailed review of cause attribution is also required for hospital deaths [28], which are also poorly certified as to cause at initial death certification (>25 % ill-defined), according to the findings from Table 4. The findings from such research would strengthen the use of TURKSTAT mortality data for descriptive epidemiology, clinical research, and health policy evaluation.

### Are we there yet?

This article describes the impressive developments in the quality of Turkish mortality statistics following the design and implementation of reforms in 2009. The TURKSTAT online database currently provides data on mortality by age and sex at the national level and for each province, for the period 2009–2013; along with data on causes of death aggregated by age and coded to a selected list of ICD-10 categories, at the national level Currently, TURKSTAT has started to compile data from death certificates in electronic formats since the beginning of 2013, and this is likely to enhance the timeliness as well as overall quality of mortality statistics.

However, there are three aspects of data quality that need to be evaluated, and if necessary, to be strengthened, before the Turkish mortality statistics system can be deemed to be of adequate functionality. Firstly, the completeness of TURKSTAT death registration at levels of around 95 % needs to be confirmed, with consensus among national and international agencies on the estimates of key summary mortality indicators including life expectancy at birth and risks of child and adult mortality. If necessary, additional research using dual record systems and capture-recapture methodology could be used as an alternate assessment of completeness for comparison. Secondly, the validity of registered causes of death needs to be established, through well-designed epidemiological studies to confirm or correct the observed cause of death patterns, as required. Finally, the assessment of data quality and estimation of mortality indicators should also be conducted at sub-national levels, to identify differentials in mortality levels, trends, and cause patterns. Such sub national assessments will improve the potential for data use, as well as identify provinces which require additional attention for system improvement.

## Conclusions

This article also presents the various steps that comprise death registration reforms in Turkey, as an example for other countries with dysfunctional vital registration and statistics systems. The reforms included the redesign of the death certificate to meet current international standards, and revised norms for data compilation, including collaboration across different stakeholders. This final step, in reporting and dissemination of positive findings as well as aspects that still need to be resolved or need further attention is necessary to focus attention on the initiatives that need to be undertaken for overall improvement in mortality data quality. This process of operational research on death registration reforms can serve as a guide to other countries faced with the need to improve the functional status of death registration.

## Additional files

Additional file 1:
**The former TURKSTAT Death Certificate.**
Additional file 2:
**The revised TURKSTAT Death Certificate.**
Additional file 3:
**The chronological development of death registration practices of TURKSTAT-DRS.**

## Abbreviations

ABPRS: Address Based Population Registration System
GBD: Global Burden of Disease
GGB: The Generalized Growth Balance Method
ICD-10: The Tenth Revision of the International Statistical Classification of Diseases and Related Health Problems
MERNIS: The Central Population Administrative System
TURKSTAT: Turkish Statistical Institute
TURKSTAT-DRS: The Turkish Statistical Institute Death Reporting System
SEG: The Synthetic Extinct Generations method
WHO: World Health Organization

## Footnotes

1 Details obtained through personal communication between the author (RO) and the Izmir Provincial Health Directorate, April 2015

2 This estimate of 91 % was observed from ‘Completeness’ tab for Turkey on the ‘GBD Mortality data visualization’ webpage; available from http://vizhub.healthdata.org/mortality/; accessed on 26 Apr 2015
