# Supplementary material for: Reliable mortality statistics for Turkey: Are we there yet?
Source: BMC Public Health. 2015 Jun 10;15:545. doi: 10.1186/s12889-015-1904-1 (PMC4460716; doi:10.1186/s12889-015-1904-1)
Supplement: Additional file 1: — The former TURKSTAT Death Certificate. [file 12889_2015_1904_MOESM1_ESM.pdf]

Additional file 1 The former TURKSTAT Death Certificate

COUNTERFOIL

Note: To be retained in responsible institution

No:……………………

I. Place where the form in filled out:

a) Province: ……………………………….

b) District: …………………………………

II. Deceased:

a) Name: …………………………………..

b) Surname: ……………………………….

c) Father's name: …………………………

d) Mother's name: ………………………...

e) Age (Completed): ……………………...

f) Sex: ………………………………………

g) House address: ……………………….. ……………………………………………… …………………………………………....

h) Cause of death (the main cause): ………………………………………………

ı) Date of death: **……./………/…………**

III. Doctor verifying death:

a) Name: …………………………………..

b) Surname: ……………………………….

Burial of deceased, name and identity written above, is permitted.

……./………/…………
Official seal and signature

REPUBLIC OF TURKEY
PRIME MINISTRY
STATE INSTITUTE OF STATISTICS

To be send to the State Institute of Statistics through Health Directorate in the province centres and Health Clinic in the district centres

DEATH STATISTICS FORM

I. Place of death

a) Name of province: ………………………………

b) Name of district: ………………………………...

II. Deceased:

a) Name and surname: …………………..………….

b) Age (completed): …. ……………………years old

c) Under one years old: ………………....month old

d) Under one month old: …………………..days old

e) Sex ◻ Male ◻ Female

f) Permanent residence: ………………… ………
◻ Province centres ◻ District centres ◻ Sub district or village

g) Marital status
◻ Never married ◻ Married ◻ Widowed ◻ Divorced

h) Education level:
◻ Illiterate ◻ Literate without a diploma ◻ Primary school
◻ Secondary school and equivalent ◻ High school ◻ University

ı) Occupation or job: ……………..…..……………
◻ House wife ◻ Retired ◻ Student ◻ Income owner ◻ Other

k) The month in which death occurred
◻ January ◻ February ◻ March ◻ April
◻ May ◻ June ◻ July ◻ August
◻ September ◻ October ◻ November ◻ December

l) Underlying cause of death: ………………………………………………………..

m) Autopsy ◻ is done ◻ is not done

III. Death verified by

◻ Hospital or health directorate ◻ Health centre physician ◻ Municipality physician

……./………/………

Official seal and signature

REPUBLIC OF TURKEY
MINISTRY OF HEALTH AND
SOCIAL WELFARE

To be given to the relatives of dead

BURIAL LICENCE

No of counterfoil: ………………….

I. Place where the form in filled out:

a) Province: ………………………..

b) District: ………………………….

II. Deceased:

a) Name: …………………………..

b) Surname: ……………………….

c) Father's name: …………………

d) Mother's name: ………………...

e) Age (Completed): ……………..

f) Sex: ………………………………

g) House address: ……………….. …………………………………………… …………………………………

h) Cause of death (the main cause): ……………………………

ı) Date of death: **…../……/………**

III. Doctor verifying death:

a) Name: …………………………..

b) Surname: ………………………

Burial of deceased, name and identity written above, is permitted.
