# Supplementary material for: Reliable mortality statistics for Turkey: Are we there yet?
Source: BMC Public Health. 2015 Jun 10;15:545. doi: 10.1186/s12889-015-1904-1 (PMC4460716; doi:10.1186/s12889-015-1904-1)
Supplement: Additional file 2: — The revised TURKSTAT Death Certificate. [file 12889_2015_1904_MOESM2_ESM.pdf]

Additional file 2 The revised TURKSTAT Death Certificate

DEATH CERTIFICATE

S1 Form no: 0000000001

Province ……………………………………………………☐☐ **Town/Village** …………………………………☐☐

District ……………………………………………………☐☐ **Institute** …………………………………☐☐

A DECEDENT'S INFORMATION

Identity (ID) Number ☐☐☐☐☐☐☐☐☐☐☐☐
☐ No ID

Nationality ☐TR
☐Other……………☐☐☐☐

Name and Surname ……………………………………………
Father's Name ……………………………………………..
Date of Birth (Day/Month/Year) ☐☐ ☐☐ ☐☐☐☐
Place of Birth …………………………………
Volume number (In ID Card) ☐☐☐☐ Family sequence number (In ID Card) ☐☐☐☐☐
Person sequence number (In ID Card) ☐☐☐☐☐

Sex ☐Male ☐Female
Education level (Completed) …………………………… ☐☐
Profession ………………………… ☐☐☐☐

Residence:

Province …………………………… ☐☐
District …………………………… ☐☐
Town or Village …………………………… ☐☐
Abroad …………………………… ☐☐☐☐

B

Date of Death (Hour/Day/Month/Year) ☐☐ ☐☐ ☐☐ ☐☐☐☐
Place of Death
☐Decedent's Home ☐Hospital ☐Work Place
☐Ambulance ☐Other vehicles ☐Other

C MANNER OF DEATH

☐Natural ☐Traffic accident ☐Other
☐Homicide ☐Industrial accident ☐Pending investigation
☐Suicide ☐Other accidents ☐Could not be determined

D Did the death occurr as a result of injury?

☐Yes ☐No --------------- ► **Go to section E**
Did injury occur in the workplace ☐Yes ☐ No
Date of injury (Day/Month/Year) ☐☐ ☐☐ ☐☐☐☐

Place of injury
☐Decedent's Home ☐Sports area
☐Residential institution ☐Street and highway
☐Rural area (farm) ☐Trade and service area
☐Industrial and const. ☐School or other Ins.
☐Other *(explain)* ……………………………………………………………

E **Was an autopsy performed**?

☐Yes ☐No ──► **Go to section F**
Were autopsy findings available to complete the cause of death?
☐Yes ☐No ──► **Go to section F**
Can more information be obtained later?
☐ Yes ☐ No

INFORMANT
Name and Surname:
Phone :
Relationship to decedent:
Signature :

CERTIFYING PHYSICIAN
Name and Surname :
Title:
Date:
Signature:
License number:

F **Stillbirth** ☐Yes ☐No
Infant mortality ☐Yes ☐No } **Go to section G**
Mother's ID No: ☐☐ ☐☐☐☐☐☐☐☐☐
Mother's age ☐☐ Number of birth☐☐
Time of Delivery ☐☐
Gestation ☐☐ Birth weight☐☐☐☐

G If Female: ☐Pregnant at time of death ☐Death occurred during delivery ☐Not pregnant, but pregnant with in 42 days of death
☐Not pregnant, but pregnant 43 days to 1 year before death ☐No maternal death

H

**PART I**	**CAUSE OF DEATH**	**Approximate interval: Onset to death**
Enter the chain of events-diseases, injures, or complications- that directly caused the death*	a)………………………………………………………………… Due to (or as a consequence of)	……………………………………………
**Immediate cause:** Final disease or condition resulting in death.	b)………………………………………………………………… Due to (or as a consequence of)	……………………………………………
Sequentially list conditions, if any, leading to the cause listed on line a.	c)………………………………………………………………… Due to (or as a consequence of)	……………………………………………
Enter the **Underlying cause** (disease or injury that initiated the events resulting in death) last	d)…………………………………………………………………	……………………………………………
**PART II**		
Enter other significant conditions to death but not resulting in the underlying cause given PART I.	…………………………………………………………………	……………………………………………
	…………………………………………………………………	……………………………………………

**Do not enter terminal events such as cardiac arrest or respiratory arrest without the etiology.*
