# Supplementary material for: Reliable mortality statistics for Turkey: Are we there yet?
Source: BMC Public Health. 2015 Jun 10;15:545. doi: 10.1186/s12889-015-1904-1 (PMC4460716; doi:10.1186/s12889-015-1904-1)
Supplement: Additional file 3: — The chronological development of death registration practices of TURKSTAT-DRS. [file 12889_2015_1904_MOESM3_ESM.pdf]

Additional file 3 The chronological development of death registration practices of TURKSTAT-DRS

Years	The development of TURKSTAT-DRS
**1931**	TURKSTAT started to collect cause of death statistics
**1931-1949**	TURKSTAT collected cause of death statistics for most populous 25 provincial centers.
**1950-1956**	TURKSTAT collected cause of death statistics for all provincial centers.
**1957**	The coverage of cause of death statistics were expanded to all provincial and district centers.
**2003**	Upgrading the Statistical System of Turkey Program cooperation with European Statistical Office (Eurostat) was started.
**2005**	The Law of Turkish Statistical Institute (Law No: 5429) was constituted in order to establish TURKSTAT's duties and responsibilities.
**2008**	The TURKSTAT Death Certificate has been adapted to international standards for recording multiple causes of death. All physicians across the country were trained about filling the TURKSTAT Death Certificate and the functioning of the system by the end of 2008. The reference physicians who are responsible for data quality from each province and each health institution were selected. The reference physicians are obliged to check the quality of data and the continuity of training.
**2009**	Reformed TURKSTAT-DRS has started to implement. ICD-10 instead of ICD-8 was adopted in the coding of cause of death. Death records collected by the MERNIS and TURKSTAT-DRS has started to reconcile.
**2013**	TURKSTAT started to compile data from death certificates in electronic formats.

Source: Turkish Statistical Institute
